# Genome wide association analysis of cuticle deposition in laying hens

**DOI:** 10.1016/j.psj.2023.102990

**Published:** 2023-08-08

**Authors:** Zhang Wang, Ian C. Dunn, Peter W. Wilson, Sandra Poyatos Pertinez, Janet E. Fulton, Jesus Arango, Björn Andersson, Matthias Schmutz, Anna Wolc

**Affiliations:** ⁎ The Roslin Institute and Royal (Dick) School of Veterinary Studies, University of Edinburgh, Edinburgh EH25 9RG, United Kingdom; † Hy-Line International, Dallas Center, IA, USA; ‡ Lohmann Breeders GmbH, Cuxhaven, DE 27472, Germany; § Department of Animal Science, Iowa State University, Ames, IA, USA

**Keywords:** egg quality, genetics, zoonoses, candidate genes, vertical transmission

## Abstract

The cuticle is an invisible barrier that protects the internal egg contents from microorganisms entering through gas exchange pores. Eggs which have a good cuticle are least likely to be penetrated by microorganisms and improved cuticle cover should reduce vertical transmission of microorganisms and improve biosecurity. The aim was to carry out a genome wide association study for cuticle deposition in 3 independent populations of laying hens using tartrazine and lissamine green staining. Eggs from ∼8,000 hens represented 2 White Leghorn and 1 Rhode Island Red breed. Estimates of heritability using pedigree or genomic relationship matrices were in the 0.2 to 0.3 range.

The results were breed specific. Across the populations, genomic regions on chromosomes 1, 2, 4, 5, and 8 were identified as significantly associated with cuticle deposition. No single loci had a large effect. A comparison was made with genes differentially expressed in the shell gland when cuticle deposition was manipulated, however none were obvious candidates for cuticle deposition. The results support the polygenic nature of the trait and the information will help in the future to understand the genetic variance and what might control cuticle deposition and the microbiological safety of the egg.

## INTRODUCTION

The cuticle on domestic hen's egg is essentially invisible. It forms a gas permeable barrier layer on the exterior of the egg (Gilbert et al., 1971; Kulshreshtha et al., 2022). The cuticle prevents the movement of particles including microorganisms across the eggshell through the gas exchange pores (Board and Halls, 1973). Within the natural variation found in a population of hens, it has been possible to demonstrate that the more cuticle deposited the greater the likelihood that no bacteria will enter the egg (Bain et al., 2013, 2019). The role of the cuticle in protecting the egg after oviposition from environmental contamination is also evident in that the cuticle of birds that nest in dirtier environments are usually much thicker (von Nathusius, 1893; Kusuda et al., 2011; D'Alba et al., 2014, 2016). Furthermore, many of the cuticle proteins are known to have antimicrobial activity, indicating their important role in protecting the egg contents (Wellman-Labadie et al., 2008; Rose-Martel et al., 2012; Bain et al., 2013). Although it is a practice in some countries to wash eggs prior to sale, it has been shown that cuticle is still present in the eggshell pores, and thus can still provide microbial protection (Kulshreshtha et al., 2018). Although there is much to still learn about the cuticle, we know that the cuticle is deposited in the very final stages before oviposition and that stimulation of the oviposition by the proximal endocrine cues, such as vasotocin or prostaglandin, result in eggs with no cuticle (Wilson et al., 2017). The principle constituents of the cuticle with the systematic nomenclature in brackets are Ovocleidin-116 (MEPE), ovocalcyxin-32 (RARRES1), ovocalyxin-36 (BPIFB3), whey acidic protein (WAP), ovocleidin-17, and clusterin (CLU) (Bain et al., 2013) backed up by transcriptomics (Pertinez et al., 2020) although many other minor proteins have been detected (Rose-Martel et al., 2012).

We have previously demonstrated that cuticle deposition has a strong genetic determination with moderate heritability of around 0.38 in a meta-analysis across breeds (Dunn et al., 2019). Beyond that, it has been difficult to determine what other factors control the deposition of the cuticle. Mild stress was identified as a factor that decreased cuticle deposition, although the effect was small in comparison with the genetic effect (Wilson et al., 2017). Hen age, which has effects on most eggshell parameters, was not a factor in our previous studies when the same hens were followed through a laying cycle (Bain et al., 2019). However, there are reports of compositional changes including glycosylation over the laying cycle (Rodriguez-Navarro et al., 2013) and in a study of different commercial breeds one saw decreased cuticle deposition up to 60 to 70 wk of age, one was not significantly different and a third increased (Sirri et al., 2022).

Understanding what controls the deposition of the cuticle and thus the potential of genomic selection can help strengthen the protection the cuticle offers to the egg content from harmful micro-organisms. The transmission of bacteria from parent to offspring is an obvious aspect of biosecurity that the cuticle can improve for the poultry industry. In chickens such vertical transmission, as well as horizontal transmission from the environment, can also threaten embryo development (Sparks and Burgess, 1993; Barrow, 1994; Stanley et al., 2003).

Although the poultry industry already has an advantage with the separation of the mother and offspring due to artificial incubation, there is still the potential for exposure of the egg to microorganisms while being laid and from the environment before the egg is collected for incubation.

Thus, the aim of the current study was to discover if there were genomic loci that were responsible for a significant component of the genetic variance for cuticle deposition that might lead to understanding what controls the deposition of the cuticle. It was hoped that the genes identified from comparing hens where the cuticle deposition was manipulated hormonally might be colocated in these regions (Pertinez et al., 2020). However, It should be remembered that no differentially expressed genes were detected in the shell gland when hens with good and poor cuticle were compared (Pertinez et al., 2020). In this study, 3 separate genetic breeds were examined comprising around 8,000 individuals in total. We used the staining protocol which we have used previously (Dunn et al., 2019) which is specific to the cuticle (Brackenridge, 1960) and is related to the cuticle depth (Chen et al., 2019). However, unlike in previous studies the “L*” from the Lab measurements from a colorimeter was used to quantify cuticle staining for some of the observations. We had demonstrated this was the same as reflectometry at a wavelength of 640 nm (Dunn et al., 2019).

## MATERIALS AND METHODS

This study did not involve experimentation on animals only the measurement of eggs.

**Figure 1:**
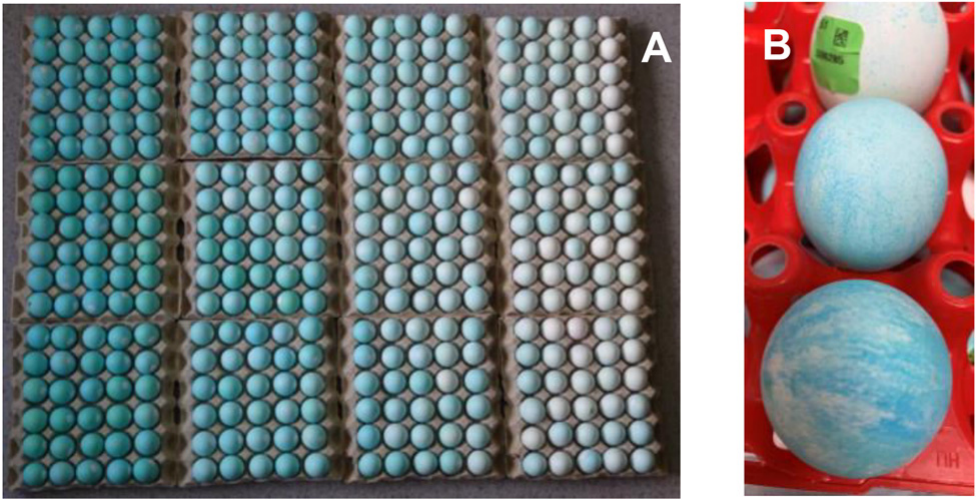
(A) Eggs from WL^2^ arranged in rows from the most cuticle staining on the left to the least on the right. (B) An example of eggs with uneven or marbled staining from WL^1^.

Three pure breeds were evaluated for cuticle deposition using tartrazine and lissamine green staining (Bain et al., 2013). The breeds are part of the breeding program for the 4 way crosses used to produce the final product for the laying hen market. In the case of breed RIR it is a Rhode Island red breed used in the production of the Hy-line brown commercial egg layer, WL^1^ was a White Leghorn breed contributing to the Hy-line W-36 commercial egg layer, and WL^2^ is also a White Leghorn breed contributing to the Lohmann LSL commercial egg layer. One egg was collected from each hen at 43 to 50 wk of age for RIR, 47 to 54 wk of age for WL^1^ and 2 eggs at 46 wk of age for WL^2^. Changes in cuticle deposition with age were not observed previously when individual hens were followed (Bain et al., 2019) although in a study of different commercial breeds a range of outcomes were observed, in one breed there was decreased cuticle deposition up to 60 to 70 wk of age, one was not significantly different and a third increased (Sirri et al., 2022). Eggs showing an irregular pattern of staining, for example Figure 1B, were removed from the analysis. These were relatively common only in the RIR where 29% of eggs were removed but only 3% from the WL^1^ and none were removed from the WL^2^ breed eggs. Eggs were only measured at one site on the equator for WL^1^, RIR, and for WL^2^ per egg as previous studies had shown that within egg variance was only around 1.4% of the total variance (Bain et al., 2013). One egg per hen was used to estimate genetic parameters for WL^1^ and RIR and the mean of the two eggs for WL^2^.

For measurement of the cuticle, eggs were submerged in the lissamine green/tartrazine solution for 30 s, rinsed, dried overnight and either the poststaining color on L*a*b scale was read using a Minolta 400 CR (breed WL^1^) or measurement was made using the Ecutimeter 2 (breed WL^2^) as previously described using the difference in absorbance at 640 nm (ΔAbs@640 nm) (Dunn et al., 2019). For brown eggs (breed RIR) both pre- and poststaining color was recorded and cuticle deposition was estimated using the following formula:

$$\Delta E^{*}\;\text{ab}\;=\;\surd \left[{\left(\Delta L*\right)}^{2}\;+\;{\left(\Delta a*\right)}^{2}\;+\;{\left(\Delta b*\right)}^{2}\right]$$

For white eggs a previous study (Dunn et al., 2019) showed a very strong correlation (−0.93) between poststaining L*a*b value and the full formula. Therefore, for white eggs from breed WL^1^ only poststaining color measurement value was taken and used as an estimate of cuticle deposition and the L* value was used to estimate the intensity of staining as we had also shown that it was highly correlated (*r*^2^, 0.995) with absorbance at 640 nm (Dunn et al., 2019)

Rhode Island Red (**RIR**, *n* = 3,073), White Leghorn (**WL^1^**, *n* = 4,322), and White Leghorn (**WL^2^**, *n* = 960) hens with cuticle phenotypes were genotyped on separate breed-specific Axiom 54k SNP chips. Quality control using 5% MAF and 90% call rate performed in PLINK (Purcell et al., 2007) resulted in 46,612, 28,345, and 21,377 segregating SNPs in the RIR, WL^1^ and WL^2^ breeds respectively. Principal component analysis was also performed in PLINK and the first 3 components were fitted as covariates in a linear mixed model in GCTA (Yang et al., 2011) together with fixed effect of hatch. Variance components and SNP effect estimates were generated from the model.

y=μ+PCA1+PCA2+PCA3+hatch+Animal+e

To estimate the number of independent SNPs for multiple testing correction pairwise pruning was performed in Plink with 500 kb windows, sliding by 10 SNPs and *r*^2^ of 0.2. In the RIR breed 1470, in the WL^1^ breed 676 and in the WL^2^ breed 810 SNPs were found to be independently segregating resulting in an adjusted *P* value threshold of 3.40 × 10^−05^, 7.40 × 10^−05^, and 6.17 × 10^−05^ for genome wide significance at 0.05 level and suggestive threshold 0.1/nSNP of 6.80 × 10^−05^ 1.48E^−04^ and 1.23 × 10^−04^.

Data were visualized as Manhattan plots using qqman package (Turner et al., 2018) in R (R Core Team, 2022). Chicken genome GalGal6a SNP locations were used. Genes within 1 Mb from the most significant SNPs were identified using ensemble genome browser (Cunningham et al., 2022).

## RESULTS

The data on cuticle deposition are summarized in Table 1.

**Table 1:** Summary statistics for cuticle deposition.

Breed	Age (wk)	*N*^1^	Average cuticle deposition^2^	SD^3^	CV^4^ (%)	Min	Max
RIR	43–50	3073	13.66	6.62	48	0.17	29.85
WL^1^	47–54	4322	82.26	4.00	5	64.41	95.75
WL^2^	46	985	0.239	0.003	1	0.002	0.547

1 Number of hens sampled.

2 The average cuticle deposition data for the RIR breed were from the L*a*b-derived ΔE∗ab units from a Minolta colorimeter and for WL^1^ breed were the difference in L* values from a Minolta colorimeter while WL^2^ values were the difference in absorbance at 640 nm using the Ecutimeter.

3 Standard deviation.

4 coefficient of variation.

The mean ± SD L*a*b* values for RIR prestaining were L*, 62.05 ± 3.511; b* 29.93 ± 2.01; a*, 16.53 ± 4.91 and poststaining L*, 57.04 ± 4.92; b* 23.93 ± 3.70; a*, 4.82 ± 6.61; for WL1 poststaining L*, 82.17 ± 3.97; b*, −9.82 ± 3.45; a*, −17.30 ± 5.49. For WL2 there was no L*a*b* values since only an absorbance at 640 nm was measured.

**Figure 2:**
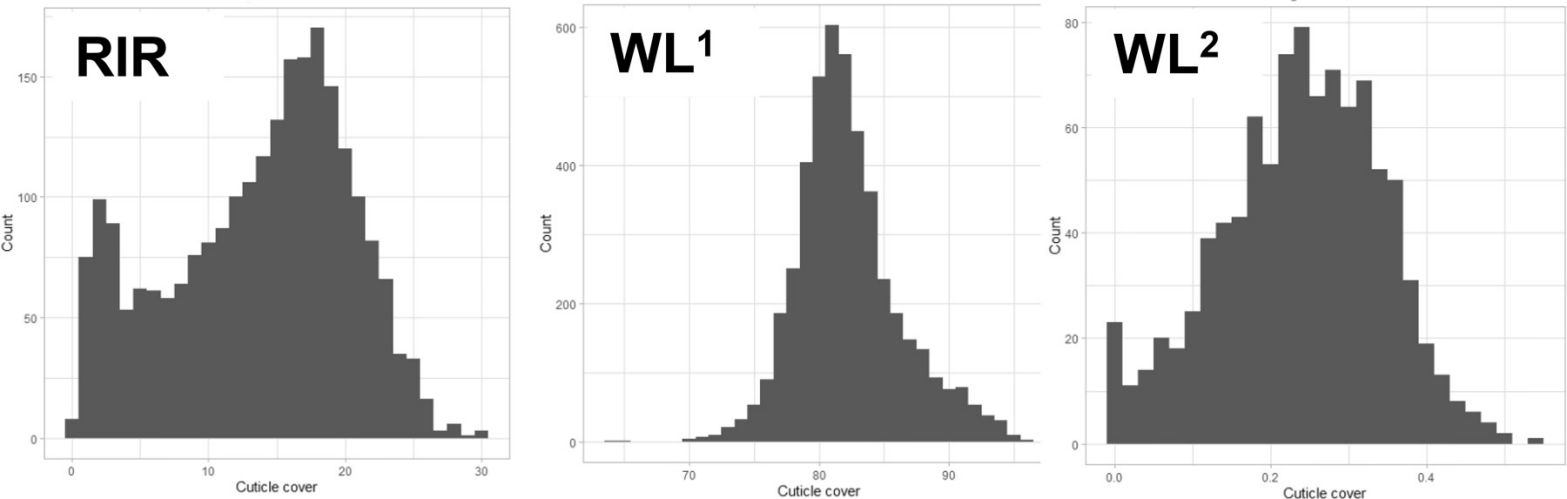
The distribution of estimated cuticle deposition. The *x*-axis is the interval of cuticle deposition. For breed RIR in L*a*b-derived ΔE∗ab units; breed WL^1^ L*a*b units and for WL^2^ ΔAbs@640 nm units. The *y*-axis represents the number of birds in each interval.

In all breeds the estimated cuticle deposition approximately followed a normal distribution (Figure 2). A picture of eggs from WL^2^ showing the variation with eggs ranging from little cuticle deposition through to strong cuticle deposition on the left emphasizes the variation present (Figure 1A). The average standard deviation of the 2 eggs measured for WL^2^ was 0.050 and the *r*^2^ of a regression of the 2 eggs was 0.44.

In all breeds estimates of genomic heritability were significantly different from zero, with the highest estimate being in WL^2^ breed (*h*^2^ = 0.35) followed by the RIR breed (*h*^2^ = 0.20) and finally the WL^1^ breed (*h*^2^ = 0.14). Variance components are given in Table 2. The estimate of heritability derived from pedigree data with hatch as a fixed effect was *h*^2^ = 0.38 ±0.08 for the WL^2^ breed, *h*^2^ = 0.28 ± 0.04 for the RIR breed and *h*^2^ = 0.19 ± 0.03 for the WL^1^ breed.

**Table 2:** Estimates of variance components and respective standard errors.

	RIR		WL^1^		WL^2^	
Source	Variance	SE	Variance	SE	Variance	SE
*V*_g_	8.97	1.51	2.29	0.32	0.0039	0.0007
*V*_e_	35.22	1.43	13.82	0.33	0.0071	0.0005
*V*_p_	44.19	1.53	16.11	0.39	0.0110	0.0006
*h*^2^	0.20	0.03	0.14	0.02	0.35	0.05

*V*_g,_ genetic variance or variance explained by markers; *V*_e_, residual variance; V_p_, phenotypic variance; *h*^2^, heritability.

### Genome Wide Association Analysis

**Figure 3:**
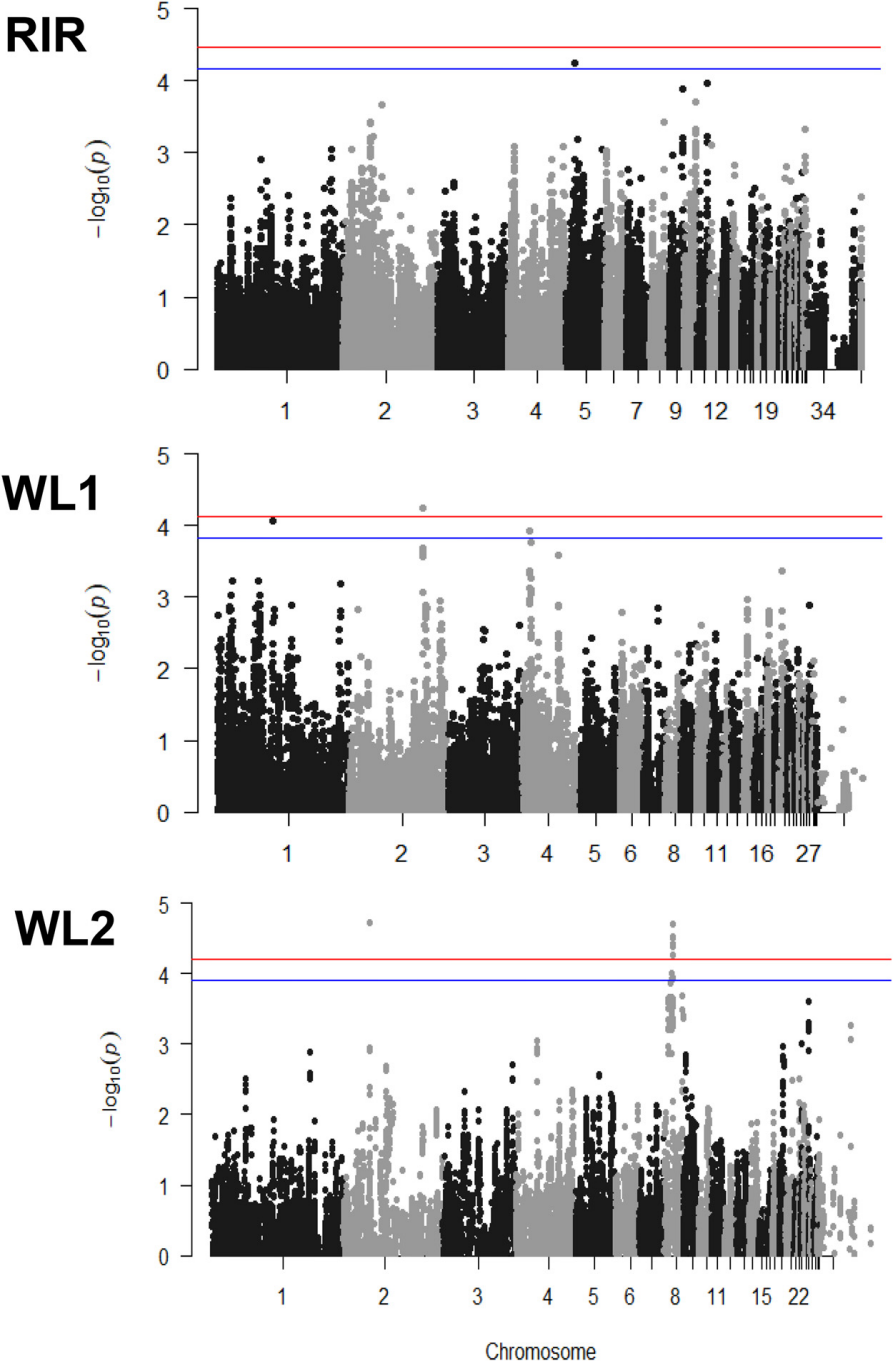
Genome wide association in RIR, WL^1^, and WL^2^ breeds; red line represents genome wide significant threshold of 0.05, blue line represents genome wide suggestive threshold of 0.1.

A summary of genome wide association analysis (**GWAS**) results can be found in Table 3 and Figure 3. In total, 9 SNPs were considered significant at a multiple testing adjusted alpha of 0.05. These were distributed across 3 genomic regions on 2 chromosomes (chromosome 2 and 8) in the WL^1^ and WL^2^ breed. Of these, 7 SNPs were on chromosome 8 in the WL^2^ breed. Further, 7 SNPs were considered suggestive at a multiple testing adjusted alpha of 0.1. These were distributed across 4 genomic regions on 4 chromosomes (chromosome 1, 5, 4, and 8) in the RIR, WL^1^, and WL^2^ breeds. Of these, 4 SNPs were associated with the chromosome 8 locus in the WL^2^ breed.

**Table 3:** Summary of the genome wide associations considered significant at *P* < 0.05 and *P* < 0.1 for RIR, WL^1^ and WL^2^.

Breed	Chromosome	Marker name	*P* value	Top SNP location (bp)	MAF*	Top SNP effect (SE)	PVE⁎⁎
RIR	5	AX-249862550	5.57E-05	14339488	0.43	0.289	0.016
WL^1^	1	AX-75530388	8.45E-05	82796729	0.11	0.175	0.016
WL^1^	**2**	AX-75967370	**5.65E-05**	**110496006**	**0.38**	**0.109**	**0.017**
WL^1^	4	AX-76609291	1.21E-04	13057383	0.05	0.257	0.015
WL^2^	**2**	AX-76089627	**1.89E-05**	**40625240**	**0.43**	**0.0057**	**0.019**
WL^2^	8	AX-77058972	1.00E-04	12059382	0.18	0.0089	0.016
WL^2^	8	AX-77059306	1.16E-04	12195775	0.17	0.0089	0.015
WL^2^	**8**	AX-77063518	**3.17E-05**	**13936981**	**0.37**	**0.0064**	**0.018**
WL^2^	**8**	AX-77063614	**5.41E-05**	**13982349**	**0.17**	**0.0089**	**0.017**
WL^2^	**8**	AX-77063615	**4.06E-05**	**13982404**	**0.34**	**0.0065**	**0.017**
WL^2^	**8**	AX-77063903	**3.03E-05**	**14104538**	**0.34**	**0.0065**	**0.018**
WL^2^	**8**	AX-77063978	**4.12E-05**	**14128407**	**0.17**	**0.0089**	**0.017**
WL^2^	**8**	AX-77064140	**3.73E-05**	**14187043**	**0.34**	**0.0065**	**0.017**
WL^2^	8	AX-77064462	1.15E-04	14322390	0.35	0.0064	0.019
WL^2^	8	AX-77064485	1.20E-04	14354520	0.17	0.0089	0.015
WL^2^	**8**	AX-77064572	**1.98E-05**	**14311065**	**0.34**	**0.0065**	**0.015**

Text in bold indicates significant at *P* < 0.05 those not in bold indicates significant at the suggestive *P* < 0.1.

⁎ Minimum allele frequency.

⁎⁎ Percentage of variance explained.

The most significant SNP was on chromosome 2 (AX-76089627) in the WL^2^ breed (Figure 3 and Table 3). This SNP explains the highest amount of phenotypic variation (PVE, 0.019).

A region on chromosome 8 in the WL^2^, breed contains a number of significant markers (Figure 3 and Table 3), the most significant being AX-77064572 which is at the distal end of the region (chr8: 14311065). A further 6 significant SNPs were clustered together on chromosome 8 (chr8: 13936981–14187043). Haplotype analysis of the markers on chromosome 8 suggested 2 loci might be present. One block contained 2 SNPs at genomic region 8a (Table 3) and was 482 kb in length (∼11.75–12.23 Mb). The second contained 9 SNPs in region 8b (Table 3) and was 499 kb in length (∼13.93–14.43 Mb).

The genes, both coding and noncoding, found in a 1 Mb region flanking the significant or suggestive SNPs are in Supplementary Table S1.

Within 1 Mb of the loci flagged as significant or suggestive there were 278 genes (Supplementary Table 1). When this was compared to a list of genes that were differentially expressed in an experiment to manipulate cuticle deposition (Pertinez et al., 2020) there were 37 genes that remained (Table 4). Those that had high FDR in the differential expression experiment were ENSGALG00000006647, ENSGALG00000006681 and ENSGALG00000006697 which were dual specificity phosphatase 8, BR serine/threonine kinase 1, and toll interacting protein respectively on chromosome 5; ENSGALG00000005340, ENSGALG00000005442, solute carrier family 35 member A3 and palmdelphin respectively on chromosome 8; and ENSGALG00000008088, acyl-CoA synthetase long chain family member 4 on chromosome 4.

**Table 4:** Summary of the genes names^3^, chromosome^1^, and position^2^ which were within 1 Mb of the GWAS positive region genes and that were differentially expressed in an experiment manipulating the deposition of the cuticle on eggs (Pertinez et al., 2020).

Gene stable ID	Chr^1^	Gene start (bp)^2^	NCBI gene^3^	*P* value^4^	FDR^5^
ENSGALG00000015094	1	83692165	Zinc finger and BTB domain containing 20	2.94E-05	3.26E-04
ENSGALG00000011442	2	39748110	Transforming growth factor beta receptor 2	9.27E-05	8.59E-04
ENSGALG00000011447	2	39825774	glutamate Decarboxylase like 1	6.88E-03	2.88E-02
ENSGALG00000011481	2	40263892	Glycerol-3-phosphate dehydrogenase 1-like	5.51E-04	3.77E-03
ENSGALG00000011518	2	41055004	Protein O-linked mannose N-acetylglucosaminyltransferase 2 (beta 1,4-)	6.52E-04	4.33E-03
ENSGALG00000015259	2	109935359	RB1 inducible coiled-coil 1	7.62E-04	4.92E-03
ENSGALG00000014432	2	111204409	Ribosomal protein S20	8.85E-03	3.52E-02
ENSGALG00000015416	2	111306864	Short-chain dehydrogenase/reductase family 16C, member 5	9.59E-04	5.93E-03
ENSGALG00000015419	2	111379997	Proenkephalin	1.34E-05	1.66E-04
ENSGALG00000007902	4	12911845	ATPase copper transporting alpha	5.14E-03	2.29E-02
ENSGALG00000008072	4	13511411	Chordin like 1	1.39E-03	8.06E-03
ENSGALG00000008088	4	13761450	Acyl-CoA synthetase Long-chain family member 4	1.07E-08	3.51E-07
ENSGALG00000008092	4	13806407	Nuclear transport factor 2 like export factor 2	3.06E-03	1.53E-02
ENSGALG00000006608	5	14310133	Cytosolic 5′-nucleotidase 1A-like	1.93E-03	1.05E-02
ENSGALG00000006647	5	14360355	Dual specificity phosphatase 8	1.94E-16	3.40E-14
ENSGALG00000006681	5	14526605	BR serine/threonine kinase 1	2.24E-06	3.57E-05
ENSGALG00000006697	5	14859903	Toll interacting protein	2.32E-06	3.68E-05
ENSGALG00000006717	5	15071724	Ovomucin, alpha subunit	2.03E-03	1.10E-02
ENSGALG00000005147	8	11450029	Amylase, alpha 1A; hepatic	1.09E-04	9.84E-04
ENSGALG00000020879	8	12141354	Solute carrier family 30 member 7	1.23E-02	4.53E-02
ENSGALG00000005257	8	12271795	Vascular cell adhesion molecule 1	5.08E-03	2.27E-02
ENSGALG00000005280	8	12304101	Dihydrolipoamide Branched chain transacylase E2	3.07E-03	1.53E-02
ENSGALG00000005290	8	12328300	tRNA methyltransferase 13 homolog	6.28E-03	2.68E-02
ENSGALG00000005302	8	12336171	SAS-6 centriolar Assembly protein	9.91E-03	3.82E-02
ENSGALG00000005340	8	12370159	Solute carrier family 35 member A3	1.31E-05	1.63E-04
ENSGALG00000005442	8	12531463	Palmdelphin	1.54E-09	6.12E-08
ENSGALG00000025896	8	12637014	Phospholipid phosphatase related 4	1.19E-02	4.41E-02
ENSGALG00000005478	8	12790282	Sorting nexin 7	1.25E-02	4.58E-02
ENSGALG00000005776	8	14137372	Trans-2,3-enoyl-CoA reductase	5.22E-04	3.60E-03
ENSGALG00000005782	8	14159785	Deoxynucleotidyltransferase, terminal, interacting protein 2	1.91E-03	1.04E-02
ENSGALG00000005782	8	14159785	Breast cancer antiestrogen resistance 3	1.91E-03	1.04E-02
ENSGALG00000005782	8	14159785	Glutamate-cysteine ligase modifier subunit	1.91E-03	1.04E-02
ENSGALG00000005850	8	14265560	Formin binding protein 1 like	9.42E-05	8.70E-04
ENSGALG00000005858	8	14328120	Downregulator of transcription 1	8.96E-07	1.61E-05
ENSGALG00000005922	8	14447758	Ribosomal protein L5	2.47E-03	1.29E-02
ENSGALG00000005977	8	14636254	BTB domain containing 8	6.60E-03	2.79E-02
ENSGALG00000006091	8	14942815	Probable ATP-dependent DNA helicase HFM1 isoform	4.71E-04	3.29E-03

The genes *P* values^4^ and the FDR^5^ from the comparative differential RNA seq analysis are presented.

## DISCUSSION

The studies demonstrated again that there was a substantial genetic component for cuticle deposition which had been shown from pedigree based heritability (Bain et al., 2013; Li et al., 2018; Dunn et al., 2019). Genetics appears to have a greater effect than any individual environmental effect such as age or stress on cuticle deposition, although clearly there are environmental factors that are yet to be discovered (Wilson et al., 2017; Sirri et al., 2022). The marker based heritability estimates for all breeds were a little lower than those reported based on pedigree, for breed WL^2^ they were moderate and similar to that previously reported (Dunn et al., 2019). The heritability estimate for WL^1^ was lower than observed previously in any breed, but the RIR breed estimate was within the range seen across layer and broiler breeds previously (Dunn et al., 2019) and both would be considered low heritability estimates.

The methods of quantifying cuticle deposition used the same staining method but the measurement of the staining differed across the breeds so it is not possible to make comparisons between breeds. They were also housed in different places so management, diets and a number of factors would not be comparable. Cuticle deposition was seen to be normally distributed although there may be a population of hens in the RIR breed which are depositing comparatively little cuticle and the number of eggs that had to be removed due to patchy deposition was also large in the RIR population. The coefficient of variance for the RIR breed was much higher at 48% compared to 5% for WL^1^ and 1% for WL^2^ which may reflect the different distribution in RIR. Where we do have data on 2 eggs for WL2, the correlation between them is certainly not close to 1 at 0.44. The use of 2 eggs may reduce some of the effects of within egg variability and might be one of the reasons the heritability is better in WL^2^, but this would need to be systematically checked. A number of eggs particularly in the RIR do show irregular patterns of staining and these may represent an increased risk of bacterial penetration which is not captured by staining and reflectometry used in this study. The hens that lay these eggs with less homogeneous cuticle represent an interesting phenomenon to pursue in an effort to try and understand the physiology and genetics of cuticle deposition which remains largely unclear. It is likely this is behind the larger coefficient of variance for this breed and will need a different approach to cuticle measurement than used in this study, perhaps following a recent paper on classifying the variability subjectively (Sirri et al., 2022).

Across the 3 breeds there was no commonality in the suggestive or significant SNP markers to reinforce or validate the results of one locus over another. In fact the significant SNPs and most of the suggestive SNPs were not informative in the other breeds. The most significant SNPs on chromosome 2 and 8 in WL^2^ breed, AX-76089627, AX-77063518, AX-77063614, AX-77063615, AX-77063903, AX-77063978, AX-77064140, AX-77064572 were not informative in the other breeds. Similarly AX-75967370 and AX-76609291 from WL^1^ on chromosome 2 and 4 and AX-249862550 from RIR chromosome 5 were not informative in the other breeds. Only AX-75530388 on chromosome 1 of WL^1^ was informative in the WL^2^ but not significant (*P* = 0.63) and not informative in the RIR breed. However, that does not mean the loci do not harbor genes or control regions that influence the variation in cuticle deposition in those breeds. Comparison of the potential candidate genes from the shell gland when cuticle deposition was manipulated (Pertinez et al., 2020) the genes with particularly high FDR in a differential expression experiment were ENSGALG00000006647, ENSGALG00000006681 and ENSGALG00000006697 which were dual specificity phosphatase 8, BR serine/threonine kinase 1 and toll interacting protein respectively on chromosome 5; ENSGALG00000005340, ENSGALG00000005442, solute carrier family 35 member A3 and palmdelphin on chromosome 8 and ENSGALG00000008088, acyl-CoA synthetase long chain family member 4 on chromosome 4. It should however be remembered that a comparison of the shell gland from the top and tail of the distribution of cuticle deposition from 1,000 hens did not produce any differentially expressed genes and the uterus/shell gland may not be where the differences underlying the genetic variation reside (Pertinez et al., 2020). Nor are any of the genes in the intervals obviously related to a published conservative list of major known cuticle proteins or top expressed genes at the time of cuticle formation (Bain et al., 2013; Pertinez et al., 2020). There is a cytochrome oxidase c, but it is not one of the highly expressed cytochrome oxidase c genes identified in the shell gland and similarly glycerol-3-phosphate dehydrogenase 1-like is not identical to the glycerol-3-phosphate dehydrogenase described previously as highly expressed in the shell gland at the time of cuticle deposition (Pertinez et al., 2020). However, genes whose products act as transcription factors such as dual specificity phosphatase 8 or BR serine/threonine kinase could have a role in increasing the activity of cells secreting the cuticle.

The only obvious egg protein on the list is ovomucin which is an albumen protein secreted in the magnum but has been reported in the organic matrix of the shell (Marie et al., 2015) and shell gland (Yin et al., 2020). Proenkephalin is also on the list of genes both in the differentially expressed list and the list of genes in regions with significant association. Proenkephalin has been enigmatically reported a number of times as being associated with the matrix of the avian egg and it was stimulated by the presence of an egg in the shell gland (Jeong et al., 2012; Brionne et al., 2014; Yin et al., 2020). Proenkephalin was also differentially expressed when cuticle deposition in the shell gland was manipulated (Pertinez et al., 2020). It is possible that an opioid signaling system is involved in uterus/shell gland function with a potential autocrine role reported in the mammalian uterus (Jin et al., 1988; Oszter et al., 2000). Although in a previous study we concluded that it was more likely involved in shell formation than the cuticle (Pertinez et al., 2020).

## CONCLUSIONS

In summary, a significant proportion (20–30%) of variation in cuticle deposition was explained by genetics, whether estimated by pedigree or markers as has been previously reported for a number of poultry breeds using slightly different measurement methods based on tartrazine and lissamine green staining. A few significant association signals were identified but no QTL with large effect were found, suggesting a polygenic nature for the trait. There are a number of leads to follow which have been discussed and a wider list of potential candidates generated that may help understand what is responsible for genetic variance in cuticle deposition and perhaps ultimately to what may control its deposition when put in the context of future studies.

For the purpose of open access, the author has applied a CC-BY public copyright license to any Author Accepted Manuscript version arising from this submission.

## Appendix Supplementary materials

Image, application 1
