# Supplementary material for: Genome wide association analysis of cuticle deposition in laying hens
Source: Poult Sci. 2023 Aug 8;102(10):102990. doi: 10.1016/j.psj.2023.102990 (PMC10458670; doi:10.1016/j.psj.2023.102990)
Supplement: Supplementary file 1 [file mmc1.pdf]

Gene stable ID	Chromosome	Gene start (bp)	NCBI gene (formerly Entrezgene) description
**Genes within 1Mb flanking**	**marker AX-75530388 (WL1 breed)**		
ENSGALG00000051879	1	81813213	reverse transcriptase
ENSGALG00000052522	1	81820856	ribonuclease H [Chlamydia abortus/psittaci]
ENSGALG00000040620	1	82041116	limbic system-associated membrane protein
ENSGALG00000047435	1	82281479	no protein associated with this gene
ENSGALG00000028743	1	82528080	LncRNA
ENSGALG00000049637	1	82867412	MiRNA
ENSGALG00000048000	1	82878193	LncRNA
ENSGALG00000015089	1	83026159	growth associated protein 43
ENSGALG00000049074	1	83050489	Pseudogene
ENSGALG00000054748	1	83092859	LncRNA
ENSGALG00000052956	1	83180722	LncRNA
ENSGALG00000051369	1	83197690	LncRNA
ENSGALG00000054115	1	83329534	LncRNA
ENSGALG00000015094	1	83692165	zinc finger and BTB domain containing 20
ENSGALG00000015117	1	83745457	dopamine receptor D3
ENSGALG00000037485	1	83770540	queuine tRNA-ribosyltransferase accessory subunit 2
ENSGALG00000053464	1	83782815	coiled-coil domain containing 191
**Genes within 1Mb flanking**	**marker AX-75967370 (WL1 breed)**		
ENSGALG00000031035	2	109465995	protein-L-isoaspartate (D-aspartate) O-methyltransferas
ENSGALG00000015254	2	109572158	ST18, C2H2C-type zinc finger
ENSGALG00000048885	2	109842961	Unknown protein coding
ENSGALG00000054269	2	109899091	LncRNA
ENSGALG00000015259	2	109935359	RB1 inducible coiled-coil 1
ENSGALG00000050073	2	110069886	LncRNA
ENSGALG00000015261	2	110084486	neuropeptides B and W receptor 1
ENSGALG00000050704	2	110097740	LncRNA
ENSGALG00000015269	2	110144374	opioid receptor kappa 1
ENSGALG00000013621	2	110240177	ATPase H+ transporting V1 subunit H
ENSGALG00000025941	2	110314102	regulator of G-protein signaling 20
ENSGALG00000015274	2	110336672	transcription elongation factor A1
ENSGALG00000048567	2	110366072	MiRNA
ENSGALG00000040421	2	110368788	lysophospholipase I
ENSGALG00000041086	2	110380891	mitochondrial ribosomal protein L15
ENSGALG00000050006	2	110415355	LncRNA
ENSGALG00000047762	2	110447606	LncRNA
ENSGALG00000051931	2	110447657	LncRNA
ENSGALG00000032994	2	110517253	SRY-box 17
ENSGALG00000051643	2	110521533	Pseudogene
ENSGALG00000031869	2	110575597	retinitis pigmentosa 1 (autosomal dominant)
ENSGALG00000054096	2	110703912	lipoxygenase homology domain-containing protein 1
ENSGALG00000047342	2	110741632	LncRNA
ENSGALG00000035429	2	110760621	XK related 4
ENSGALG00000049819	2	110996238	LncRNA
ENSGALG00000031835	2	111010166	transmembrane protein 68
ENSGALG00000030767	2	111032635	transmembrane protein 68-like

ENSGALG00000054504	2	111053852	LncRNA
ENSGALG00000015340	2	111084380	trimethylguanosine synthase 1
ENSGALG00000042321	2	111134283	LYN proto-oncogene, Src family tyrosine kinase
ENSGALG00000015404	2	111182778	kinesin family member 20A-like
ENSGALG00000014432	2	111204409	ribosomal protein S20
ENSGALG00000025635	2	111205002	small nucleolar RNA U54
ENSGALG00000042082	2	111223762	v-mos Moloney murine sarcoma viral oncogene homolo
ENSGALG00000030429	2	111230880	LncRNA
ENSGALG00000015407	2	111237295	PLAG1 zinc finger
ENSGALG00000043676	2	111284526	coiled-coil-helix-coiled-coil-helix domain-containing pro
ENSGALG00000015416	2	111306864	short chain dehydrogenase/reductase family 16C, meml
ENSGALG00000022827	2	111354977	epidermal retinol dehydrogenase 2-like
ENSGALG00000015419	2	111379997	proenkephalin
ENSGALG00000052587	2	111439200	LncRNA
**Genes within 1Mb flanking**	**marker AX-76089627 (WL1 breed)**		
ENSGALG00000048510	2	39735366	LncRNA
ENSGALG00000011442	2	39748110	transforming growth factor beta receptor 2
ENSGALG00000011447	2	39825774	glutamate decarboxylase like 1
ENSGALG00000011465	2	40078293	STT3B, catalytic subunit of the oligosaccharyltransferase
ENSGALG00000050427	2	40134661	oxysterol binding protein like 10
ENSGALG00000025305	2	40151376	microRNA 1622
ENSGALG00000011481	2	40263892	glycerol-3-phosphate dehydrogenase 1-like
ENSGALG00000011484	2	40304543	CKLF like MARVEL transmembrane domain containing 8
ENSGALG00000011488	2	40345059	CKLF like MARVEL transmembrane domain containing 7
ENSGALG00000011490	2	40384646	CKLF like MARVEL transmembrane domain containing 6
ENSGALG00000011491	2	40398740	dynein cytoplasmic 1 light intermediate chain 1
ENSGALG00000011494	2	40442858	CCR4-NOT transcription complex subunit 10
ENSGALG00000019622	2	40477423	tripartite motif containing 71
ENSGALG00000011498	2	40558064	T-cell activation inhibitor, mitochondrial
ENSGALG00000054787	2	40601273	testis and ovary specific PAZ domain containing 1 TOPA
ENSGALG00000052964	2	40639239	TOPAZ1 isoform
ENSGALG00000018283	2	40676597	microRNA 138-1
ENSGALG00000050387	2	40677931	LncRNA
ENSGALG00000040363	2	40792849	abhydrolase domain containing 4
ENSGALG00000042371	2	40838346	anoctamin 10
ENSGALG00000039920	2	40962383	SNF related kinase
ENSGALG00000047614	2	41009519	LncRNA
ENSGALG00000011518	2	41055004	protein O-linked mannose N-acetylglucosaminyltransfer
ENSGALG00000037533	2	41103488	Golgi-associated kinase 1A
ENSGALG00000043379	2	41121646	ribosomal protein L14
ENSGALG00000030493	2	41127304	ectonucleoside triphosphate diphosphohydrolase 3
ENSGALG00000030552	2	41172440	gamma-glutamylcyclotransferase
ENSGALG00000036807	2	41184789	nucleotide binding oligomerization domain containing 1
ENSGALG00000031163	2	41213767	zinc and ring finger 2
ENSGALG00000029724	2	41302040	maturin, neural progenitor differentiation regulator hor
ENSGALG00000025386	2	41308566	microRNA 1723
ENSGALG00000033379	2	41331321	ELL associated factor 1
ENSGALG00000040665	2	41350205	methyltransferase like 6

ENSGALG00000039337	2	41391821	SH3 domain binding protein 5
ENSGALG00000052604	2	41438871	calpain-7 isoform
ENSGALG00000041731	2	41440864	calpain 7
ENSGALG00000054423	2	41469751	LncRNA
ENSGALG00000047341	2	41502573	Unknown
ENSGALG00000047663	2	41515331	Unknown
ENSGALG00000035854	2	41532339	collagen alpha-6(VI) chain isoform
ENSGALG00000040917	2	41543820	phosphoinositide-3-kinase regulatory subunit 4
ENSGALG00000034340	2	41622054	ATPase secretory pathway Ca2+ transporting 1
**Genes within 1Mb flanking**	**marker AX-76609291 (WL1 breed)**		
ENSGALG00000048956	4	12767858	LncRNA
ENSGALG00000007806	4	12790680	fibroblast growth factor 16
ENSGALG00000007843	4	12808926	alpha thalassemia/mental retardation syndrome X-linke
ENSGALG00000007861	4	12887297	magnesium transporter 1
ENSGALG00000007863	4	12902246	cytochrome c oxidase subunit 7B
ENSGALG00000007902	4	12911845	ATPase copper transporting alpha
ENSGALG00000007936	4	12938400	phosphoglycerate kinase 2
ENSGALG00000052899	4	12950392	LncRNA
ENSGALG00000007952	4	12977246	angiomotin
ENSGALG00000047384	4	13050893	LncRNA
ENSGALG00000007972	4	13079135	transient receptor potential cation channel subfamily C
ENSGALG00000007976	4	13151658	putative bifunctional UDP-N-acetylglucosamine transfer
ENSGALG00000030718	4	13177222	UDP-N-acetylglucosamine transferase subunit ALG13 ho
ENSGALG00000051247	4	13181957	LncRNA
ENSGALG00000007993	4	13231208	doublecortin
ENSGALG00000008006	4	13332487	calpain 6
ENSGALG00000008058	4	13392419	p21 (RAC1) activated kinase 3
ENSGALG00000025153	4	13437062	microRNA 1606
ENSGALG00000049899	4	13466861	LncRNA
ENSGALG00000008072	4	13511411	chordin like 1
ENSGALG00000054265	4	13577487	LncRNA
ENSGALG00000008074	4	13620556	Alport syndrome, mental retardation, midface hypoplas gene 1
ENSGALG00000008076	4	13698638	transmembrane protein 164
ENSGALG00000049238	4	13737935	LncRNA
ENSGALG00000008088	4	13761450	acyl-CoA synthetase long chain family member 4
ENSGALG00000025789	4	13761996	microRNA 6614
ENSGALG00000040890	4	13800702	potassium voltage-gated channel subfamily E regulatory
ENSGALG00000008092	4	13806407	nuclear transport factor 2 like export factor 2
ENSGALG00000049549	4	13814827	LncRNA
ENSGALG00000050426	4	13826678	LncRNA
ENSGALG00000008107	4	13876602	insulin receptor substrate 4
ENSGALG00000008141	4	13903972	collagen alpha-5(IV) chain isoform
ENSGALG00000053028	4	14021011	collagen alpha-6(IV) chain isoform
**Genes within 1Mb flanking**	**marker AX-249862550 (RIR breed)**		
ENSGALG00000032687	5	13345327	pleckstrin homology like domain family A member 2
ENSGALG00000006454	5	13347574	solute carrier family 22 member 18
ENSGALG00000037138	5	13429698	potassium voltage-gated channel subfamily Q member 1

ENSGALG00000053639	5	13430488	LncRNA
ENSGALG00000006521	5	13772652	transient receptor potential cation channel, subfamily M
ENSGALG00000006530	5	13810153	tumor suppressing subtransferable candidate 4
ENSGALG00000006546	5	13821201	CD81 molecule
ENSGALG00000054379	5	13850453	tetraspanin 32
ENSGALG00000030492	5	13869949	achaete-scute family bHLH transcription factor 2
ENSGALG00000028521	5	13870257	microRNA 6642
ENSGALG00000054949	5	13883040	LncRNA
ENSGALG00000029648	5	13911444	tyrosine hydroxylase
ENSGALG00000053525	5	13911584	LncRNA
ENSGALG00000006552	5	13942581	insulin
ENSGALG00000054986	5	13947427	LncRNA
ENSGALG00000048192	5	13957865	LncRNA
ENSGALG00000035282	5	13971786	insulin like growth factor 2
ENSGALG00000051551	5	13984172	LncRNA
ENSGALG00000053027	5	14002362	LncRNA
ENSGALG00000050057	5	14103126	LncRNA
ENSGALG00000049857	5	14136126	LncRNA
ENSGALG00000006561	5	14195145	mitochondrial ribosomal protein L23
ENSGALG00000006572	5	14207186	troponin T3, fast skeletal type
ENSGALG00000006583	5	14250735	lymphocyte-specific protein 1 pseudogene 1
ENSGALG00000006591	5	14291354	troponin I2, fast skeletal type
ENSGALG00000051917	5	14296860	LncRNA
ENSGALG00000006602	5	14304943	synaptotagmin 8
ENSGALG00000006608	5	14310133	cytosolic 5'-nucleotidase 1A-like
ENSGALG00000006613	5	14320728	cathepsin D
ENSGALG00000031657	5	14337982	interferon induced transmembrane protein 10
ENSGALG00000051943	5	14338471	MiRNA
ENSGALG00000006647	5	14360355	dual specificity phosphatase 8
ENSGALG00000030499	5	14412214	MOB kinase activator 2
ENSGALG00000006681	5	14526605	BR serine/threonine kinase 1
ENSGALG00000053957	5	14832517	LncRNA
ENSGALG00000052099	5	14845557	LncRNA
ENSGALG00000006697	5	14859903	toll interacting protein
ENSGALG00000044418	5	14892238	mucin-5AC
ENSGALG00000034144	5	14925899	mucin-5AC
ENSGALG00000031737	5	14975993	mucin-5AC
ENSGALG00000048053	5	15036312	LncRNA
ENSGALG00000006717	5	15071724	ovomucin, alpha subunit
ENSGALG00000049199	5	15122121	LncRNA
ENSGALG00000053204	5	15178531	Unknown
ENSGALG00000050436	5	15212550	LncRNA
ENSGALG00000040530	5	15268800	mucin 6, oligomeric mucus/gel-forming
ENSGALG00000047684	5	15301083	mucin 6, oligomeric mucus/gel-forming
ENSGALG00000006799	5	15307816	adaptor related protein complex 2 alpha 2 subunit
**Genes within 1Mb flanking marker AX-77058972 an 77063518 (WL2 breed)**			
ENSGALG00000031129	8	10922755	hemicentin 1
ENSGALG00000045555	8	11201702	influenza virus NS1A binding protein

ENSGALG00000034230	8	11220083	SWT1, RNA endoribonuclease homolog
ENSGALG00000033077	8	11257067	tRNA methyltransferase 1 like
ENSGALG00000053443	8	11308094	LncRNA
ENSGALG00000050030	8	11312205	LncRNA
ENSGALG00000050360	8	11323913	LncRNA
ENSGALG00000048341	8	11353547	LncRNA
ENSGALG00000047688	8	11407676	LncRNA
ENSGALG00000005147	8	11450029	amylase, alpha 1A; hepatic
ENSGALG00000005162	8	11468855	RNA binding region (RNP1, RRM) containing 3
ENSGALG00000048191	8	11515298	LncRNA
ENSGALG00000046817	8	11602708	collagen alpha-1(XI) chain isoform
ENSGALG00000026650	8	11640957	microRNA 6561
ENSGALG00000005180	8	11693571	collagen alpha-1(XI) chain isoform
ENSGALG00000052635	8	11753979	ribonuclease H [Chlamydia abortus/psittaci]
ENSGALG00000005203	8	11872299	olfactomedin 3
ENSGALG00000047706	8	11951332	ribonuclease H [Chlamydia abortus/psittaci]
ENSGALG00000005208	8	12067120	sphingosine-1-phosphate receptor 1
ENSGALG00000020884	8	12080882	uncharacterized oxidoreductase ZK1290.5-like
ENSGALG00000025580	8	12119261	MiRNA
ENSGALG00000005229	8	12119346	diphthamide biosynthesis 5
ENSGALG00000020879	8	12141354	solute carrier family 30 member 7
ENSGALG00000051438	8	12167220	exostosin like glycosyltransferase 2
ENSGALG00000005248	8	12174912	cell division cycle 14A
ENSGALG00000005252	8	12236462	G protein-coupled receptor 88
ENSGALG00000050710	8	12265015	MiRNA
ENSGALG00000005257	8	12271795	vascular cell adhesion molecule 1
ENSGALG00000005277	8	12295535	RNA 3'-terminal phosphate cyclase
ENSGALG00000005280	8	12304101	dihydrolipoamide branched chain transacylase E2
ENSGALG00000005284	8	12320048	leucine rich repeat containing 39
ENSGALG00000005290	8	12328300	tRNA methyltransferase 13 homolog
ENSGALG00000005302	8	12336171	SAS-6 centriolar assembly protein
ENSGALG00000005329	8	12349410	major facilitator superfamily domain containing 14A
ENSGALG00000005340	8	12370159	solute carrier family 35 member A3
ENSGALG00000052696	8	12388820	LncRNA
ENSGALG00000046652	8	12425645	hornerin isoform
ENSGALG00000005407	8	12469818	amylo-alpha-1, 6-glucosidase, 4-alpha-glucanotransfera
ENSGALG00000005418	8	12509733	ferric chelate reductase 1
ENSGALG00000005442	8	12531463	palmdelphin
ENSGALG00000005470	8	12561720	phospholipid phosphatase related 5
ENSGALG00000025896	8	12637014	phospholipid phosphatase related 4
ENSGALG00000005478	8	12790282	sorting nexin 7
ENSGALG00000052139	8	12845856	LncRNA
ENSGALG00000054920	8	12859454	LncRNA
ENSGALG00000051416	8	12911047	LncRNA
ENSGALG00000048736	8	12920099	LncRNA
ENSGALG00000018336	8	12923059	microRNA 137
ENSGALG00000034994	8	12961396	LncRNA
ENSGALG00000005509	8	12966245	dihydropyrimidine dehydrogenase

ENSGALG00000005552	8	13336528	polypyrimidine tract binding protein 2
ENSGALG00000049893	8	13530298	LncRNA
ENSGALG00000038421	8	13715182	RWD domain containing 3
ENSGALG00000005571	8	13729738	holocytochrome c synthase
ENSGALG00000005580	8	13738852	transmembrane protein 56
ENSGALG00000005583	8	13781317	ALG14, UDP-N-acetylglucosaminyltransferase subunit
ENSGALG00000047650	8	13811577	LncRNA
ENSGALG00000005597	8	13815244	calponin 3
ENSGALG00000005610	8	13840231	solute carrier family 44 member 3
ENSGALG00000005619	8	13932222	coagulation factor III, tissue factor
ENSGALG00000005647	8	13947647	ATP binding cassette subfamily D member 3
ENSGALG00000005683	8	13997576	Rho GTPase activating protein 29
ENSGALG00000048056	8	14057781	LncRNA
ENSGALG00000005752	8	14062866	ATP binding cassette subfamily A member 4
ENSGALG00000005776	8	14137372	trans-2,3-enoyl-CoA reductase
ENSGALG00000005782	8	14159785	glutamate-cysteine ligase modifier subunit
ENSGALG00000005782	8	14159785	deoxynucleotidyltransferase, terminal, interacting prote
ENSGALG00000005782	8	14159785	breast cancer anti-estrogen resistance 3
ENSGALG00000005850	8	14265560	formin binding protein 1 like
ENSGALG00000005858	8	14328120	down-regulator of transcription 1
ENSGALG00000032319	8	14338839	retinol dehydrogenase 8 (all-trans)
ENSGALG00000005889	8	14351493	coiled-coil domain containing 18
ENSGALG00000005904	8	14378876	transmembrane p24 trafficking protein 5
ENSGALG00000043087	8	14392869	metal response element binding transcription factor 2
ENSGALG00000005918	8	14431709	family with sequence similarity 69 member A
ENSGALG00000005922	8	14447758	ribosomal protein L5
ENSGALG00000054063	8	14450908	small nucleolar RNA SNORD21
ENSGALG00000029582	8	14456259	ecotropic viral integration site 5
ENSGALG00000043650	8	14533300	growth factor independent 1 transcriptional repressor
ENSGALG00000005953	8	14565754	RNA polymerase II associated protein 2
ENSGALG00000005959	8	14599942	glomulin, FKBP associated protein
ENSGALG00000023490	8	14618965	chromosome 8 open reading frame, human C1orf146
ENSGALG00000005977	8	14636254	BTB domain containing 8
ENSGALG00000006019	8	14672386	epoxide hydrolase 3
ENSGALG00000006031	8	14698872	bromodomain testis associated
ENSGALG00000052761	8	14736278	LncRNA
ENSGALG00000006038	8	14742786	transforming growth factor beta receptor 3
ENSGALG00000054737	8	14888913	LncRNA
ENSGALG00000006051	8	14895304	cell division cycle 7
ENSGALG00000051219	8	14912442	LncRNA
ENSGALG00000053234	8	14919148	LncRNA
ENSGALG00000006091	8	14942815	probable ATP-dependent DNA helicase HFM1 isoform
ENSGALG00000050568	8	14951227	HFM1, ATP dependent DNA helicase homolog
ENSGALG00000034253	8	15007036	zinc finger protein 644
ENSGALG00000040069	8	15109602	BarH like homeobox 2
ENSGALG00000053974	8	15158223	LncRNA
ENSGALG00000051232	8	15211727	LncRNA
